# Biohydrogen production from arabinose and glucose using extreme thermophilic anaerobic mixed cultures

**DOI:** 10.1186/1754-6834-5-6

**Published:** 2012-02-13

**Authors:** Angela A Abreu, Dimitar Karakashev, Irini Angelidaki, Diana Z Sousa, M Madalena Alves

**Affiliations:** 1 Institute for Biotechnology and Bioengineering, Centre of Biological Engineering, University of Minho, 4710-057 Braga, Portugal; 2 Department of Environmental Engineering, Technical University of Denmark, Bygningstorvet 115, DK-2800, Kgs Lyngby, Denmark

**Keywords:** biohydrogen, extreme thermophilic conditions, arabinose, hydrogen partial pressure, pH, lactate

## Abstract

**Background:**

Second generation hydrogen fermentation technologies using organic agricultural and forestry wastes are emerging. The efficient microbial fermentation of hexoses and pentoses resulting from the pretreatment of lingocellulosic materials is essential for the success of these processes.

**Results:**

Conversion of arabinose and glucose to hydrogen, by extreme thermophilic, anaerobic, mixed cultures was studied in continuous (70°C, pH 5.5) and batch (70°C, pH 5.5 and pH 7) assays. Two expanded granular sludge bed (EGSB) reactors, R_arab _and R_gluc_, were continuously fed with arabinose and glucose, respectively. No significant differences in reactor performance were observed for arabinose and glucose organic loading rates (OLR) ranging from 4.3 to 7.1 kgCOD m^-3 ^d^-1^. However, for an OLR of 14.2 kgCOD m^-3 ^d^-1^, hydrogen production rate and hydrogen yield were higher in R_arab _than in R_gluc _(average hydrogen production rate of 3.2 and 2.0 LH_2 _L^-1 ^d^-1 ^and hydrogen yield of 1.10 and 0.75 molH_2 _mol^-1^_substrate _for R_arab _and R_gluc_, respectively). Lower hydrogen production in R_gluc _was associated with higher lactate production. Denaturing gradient gel electrophoresis (DGGE) results revealed no significant difference on the bacterial community composition between operational periods and between the reactors. Increased hydrogen production was observed in batch experiments when hydrogen partial pressure was kept low, both with arabinose and glucose as substrate. Sugars were completely consumed and hydrogen production stimulated (62% higher) when pH 7 was used instead of pH 5.5.

**Conclusions:**

Continuous hydrogen production rate from arabinose was significantly higher than from glucose, when higher organic loading rate was used. The effect of hydrogen partial pressure on hydrogen production from glucose in batch mode was related to the extent of sugar utilization and not to the efficiency of substrate conversion to hydrogen. Furthermore, at pH 7.0, sugars uptake, hydrogen production and yield were higher than at pH 5.5, with both arabinose and glucose as substrates.

## Background

Hydrogen is a promising renewable energy carrier that can contribute towards a low carbon economy. Fermentative hydrogen production from carbohydrate-containing feedstock, such as glucose, sucrose and starch, has been extensively studied [1,2]. However, second generation hydrogen fermentation technologies are presently emerging as promising and more cost-effective solutions [1,3].

**Figure 1:**
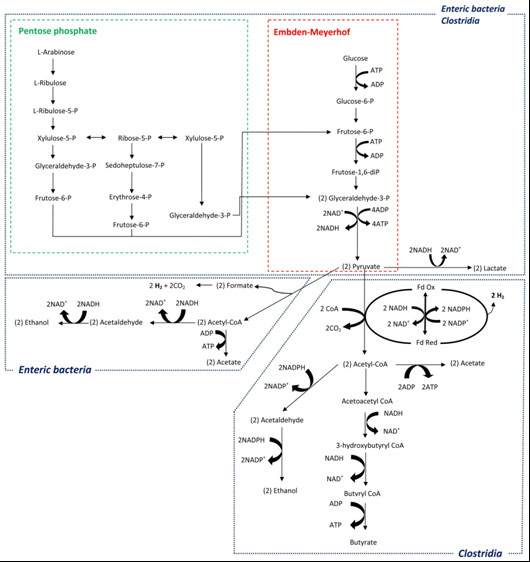
**Major metabolic pathways for glucose and arabinose fermentation in mixed cultures (adapted from **[7,8]**)**.

Lignocellulosic material must be pre-treated prior to fermentation to hydrogen in order to remove lignin and hemicelluloses, reduce the cellulose crystallinity and increase the surface area of the material to enhance the release of sugars [4]. Physico-chemical pre-treatment of lignocellulosic material, such as the application of acid, alkaline or oxidative conditions at ambient or elevated temperatures, yields a mixture of pentoses and hexoses [1]. Efficient microbial fermentation of hexoses and pentoses is, therefore, the key step for hydrogen production from plant biomass. However, combined fermentation of mixtures of hexoses and pentoses is often prevented due to catabolic repression; in the presence of glucose, pentoses might be converted to a lesser extent thereby decreasing overall fermentation yields [5,6]. Moreover, efficient hydrogen production from sugars is dependent on the different possible fermentation pathways (Figure 1).

Most of the extreme thermophiles from the phylum *Clostridia *use the Embden-Meyerhof pathway to metabolize hexose sugars to pyruvate [9]. Biohydrogen can be then formed via decarboxylation of pyruvate to acetyl CoA, in which reduced ferredoxin (Fd_red_) is generated and acts as a direct electron donor for proton reduction to hydrogen (Figure 1). Maximum hydrogen yield, both from hexoses or pentoses, is obtained with acetate as the fermentation product (equations 1 and 2). Low yields are associated with the formation of more reduced end products compared to acetate, such as butyrate, propionate and alcohols (ethanol, butanol) and lactic acid.

(1) $${\text{C}}_{\text{6}}{\text{H}}_{12}{\text{O}}_{6}+2{\text{H}}_{2}\text{O}\to \text{2C}{\text{H}}_{3}\text{CO}{\text{O}}^{-}+2\text{C}{\text{O}}_{2}+2{\text{H}}^{+}+4{\text{H}}_{2}$$

(2) $${\text{C}}_{5}{\text{H}}_{10}{\text{O}}_{5}+1.67{\text{H}}_{2}\text{O}\to \text{1}\text{.67C}{\text{H}}_{3}\text{CO}{\text{O}}^{-}+1.67\text{C}{\text{O}}_{2}+1.67{\text{H}}^{+}+3.33{\text{H}}_{2}$$

Environmental parameters such as pH, hydrogen partial pressure and temperature have been documented as key factors in hydrogen fermentation [10]. The pH of the medium is known to regulate the shift to solventogenesis during the fermentation of sugars [7]; the effect of low pH in the inhibition of methanogenic archaea is also recognized and could be potentially used as a selective pressure in mixed culture systems. Metabolic pathways of hydrogen formation are sensitive to hydrogen partial pressure (*P*H_2_) and are subject to end-product inhibition [11,12]. In addition, fermentation processes operating under thermophilic (45 to 60°C) and extreme thermophilic (65 to 80°C) could possibly result in higher hydrogen yields due to favorable thermodynamics and lower variety in soluble by-products [13]. High temperatures inhibit the growth of methanogenic archaea and homoacetogenic bacteria [13]; this is an important advantage when using mixed-cultures for hydrogen production because it prevents consumption of hydrogen by these microbial groups (as is often the case in mesophilic fermentation). Also, higher hydrolysis rates of cellulosic material have been observed in studies performed under thermophilic conditions, with the concurrent formation of higher amounts of fermentable sugars [14]. Hydrogen production by mixed culture fermentation is more suited for industrial applications, when compared to pure culture fermentation. Some of the advantages are: (i) no need for sterile cultivation, (ii) presence of high microbial diversity, which offers increased adaptation capacity, (iii) possibility of mixed substrates co-fermentation, and (iv) higher capacity for continuous processing [15,16]. However, and although there is a considerable number of studies on H_2 _production at extreme thermophilic conditions using pure cultures, studies using mixed-cultures are lacking [17,18]. Also, the effect of pH and hydrogen partial pressure has been described in several pure cultures of thermophiles and extreme-thermophiles but the effect in mixed cultures is not yet clear [17].

In the present study, the conversion of a C5-sugar (arabinose) and a C6-sugar (glucose) to hydrogen, using anaerobic mixed-cultures under extreme thermophilic conditions (70°C), was studied in continuous expanded granular sludge bed (EGSB) reactors. Microbial diversity in arabinose- and glucose-fed bioreactors was assessed using a PCR-DGGE (denaturing gradient gel electrophoresis) approach. Additional batch experiments were performed with extreme-thermophilic mixed cultures to study the effect of hydrogen partial pressure and pH on hydrogen production from arabinose and glucose.

## Results

### EGSB reactors performance

**Figure 2:**
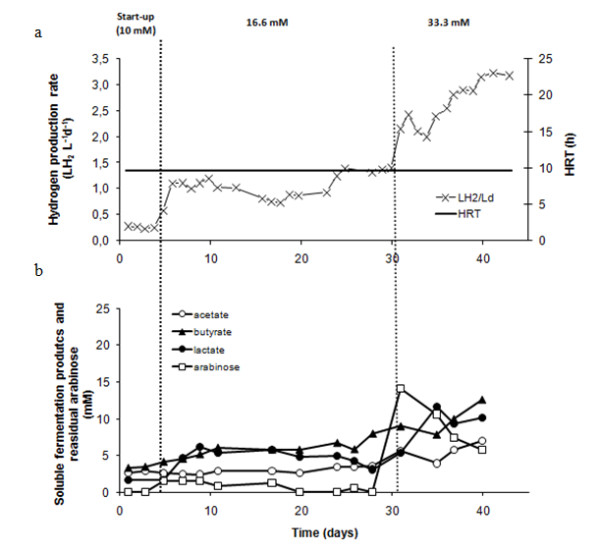
**Effect of OLR on performance of R_arab _**(a) **hydrogen production rate and HRT, **(b) **soluble fermentation products and residual arabinose**.

**Figure 3:**
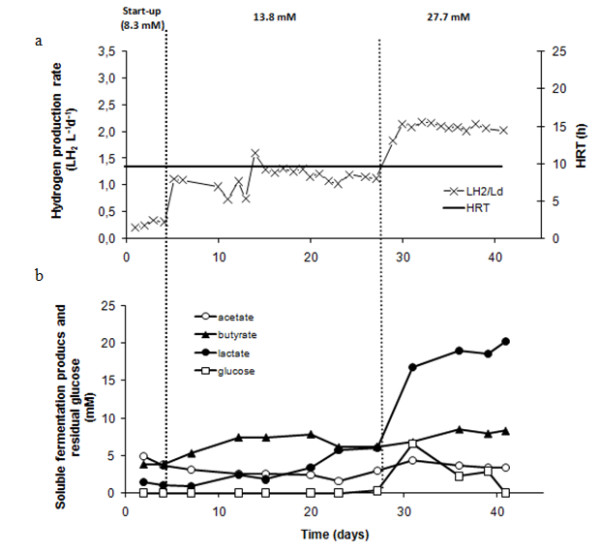
**Effect of OLR on performance of R_gluc _**(a) **hydrogen production rate and HRT, **(b) **soluble fermentation products and residual glucose**.

Hydrogen production rates in arabinose- and glucose-fed reactors (R_arab _and R_gluc_) are shown in Figures 2 and 3, respectively. Only H_2 _and CO_2 _were detected in the gas phase; methane was not produced during all operation time. During start-up (period I), hydrogen production rates of approximately 0.3 L H_2 _L^-1^d^-1 ^were observed in both reactors. This corresponds to hydrogen yields of roughly 0.2 and 0.3 mol H_2 _per mol of substrate consumed, for R_arab _and R_gluc _respectively (Table 1). In period II, the increase in arabinose and glucose inlet concentration to 16.6 mM and 13.8 mM, respectively, resulted in hydrogen yields of about 0.80 mol H_2 _per mole of substrate in both R_arab _and R_gluc _(Table 1). Maximum hydrogen production rates in period II were of 1.36 ± 0.04 and 1.12 ± 0.07 LH_2 _L^-1 ^d^-1 ^in R_arab _and R_gluc_, respectively. Substrate was completely consumed in both reactors and the main by-products formed were butyrate, acetate and lactate (Figures 2 and 3). In operation period III, substrate concentrations fed to R_arab _and R_gluc _were increased to 33.3 mM of arabinose and 27.7 mM of glucose, respectively. As a result of this increase, there was a temporary raise in arabinose/glucose concentration in the effluent but, after 13 days of acclimation to the higher substrate loads, virtually all glucose and an average of 79% arabinose were used in the reactors (Table 1) Steady state hydrogen production rates of 3.26 ± 0.16 and 2.06 ± 0.06 L H_2 _L^-1 ^d^-1 ^were observed in R_arab _and R_gluc_, respectively (Figures 2 and 3). During period III, R_gluc _showed a stable hydrogen yield of about 0.75 mol H_2 _per mole of substrate consumed. Hydrogen yield in R_arab _was significantly higher, that is. 1.10 mol H_2 _per mole of substrate consumed. Lactate concentration in R_gluc _increased sharply during period III of operation reaching values of approximately 20 mM (Figure 3). An increase in lactate concentration was also observed in R_arab_, but did not exceed 11 mM (Figure 2). Estimation of the theoretical reduced form of nicotinamide adenine dinucleotide (NADH) {AU Query: Please replace NADH in full.} production from glucose and arabinose, considering the main catabolic pathways (that is, Embden-Meyerhof for glucose and a combination of pentose phosphate and Embden-Meyerhof pathways for arabinose (Figure 1)), demonstrates that a higher reducing power was potentially formed in R_gluc _than in R_arab_. Estimated NADH concentration in R_gluc _was 42 mM after three days of operation, while in R_arab _was 37 mM after five days of operation.

**Table 1:** Process performance of R_arab_, R_gluc _and R_gluc+arab_.

	Feed concentration (mM)	Glucose utilization (%)	Arabinose utilization (%)	Hydrogen yield (molH_2 _mol substrate consumed ^-1^)	Percentage of H_2 _produced from the theoretical yield (%)	Hydrogen production rate (LH_2 _L^-1^d^-1^)	COD balance (%)*	Reference
**Glucose** **reactor** **(R_gluc_)**	8.3	100	na	0.34 ± 0.05	8	0.32 ± .018	96	This study
	13.8	100	na	0.80 ± 0.03	20	1.15 ± 0.04	94	
	27.7	100	na	0.75 ± 0.07	19	2.10 ± 0.06	99	

**Arabinose** **reactor** **(R_arab_)**	10.0	na	100	0.23 ± 0.01	7	0.24 ± 0.01	113	This study
	16.6	na	99	0.77 ± 0.02	23	1.36 ± 0.04	97	
	33.3	na	79	1.10 ± 0.01	33	3.26 ± 0.16	112	

**Glucose + Arabinose Reactor (R_gluc+arab_)**	13.8+16.6	100	75	0.77 ± 0.05	---	2.36 ± 0.14	109	[6]

*Assuming 15% of COD for cell growth.

### Bacterial community composition dynamics in EGSB reactors

**Figure 4:**
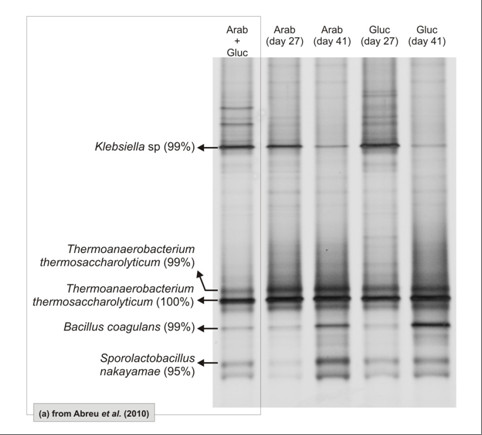
**DGGE profile of granular sludge samples from a reactor fed with arabinose and glucose **[6]**and at Day 27 and Day 41 from arabinose (and glucose reactors**.

DGGE profiles generated for sludge samples withdrawn from R_arab _and R_gluc _(Figure 4) show that bacterial composition in both reactors' sludge at the end of periods II (Day 27) and III (Day 41) are identical. Differences in substrate composition did not affect the bacterial community in reactors R_arab _and R_gluc _and similarity index between Arab/Gluc samples at the end of the operation was as high as 94%. Predominant DGGE bands in R_gluc _and R_arab _were identical to the ones present in the inoculum used in this study and for which the phylogeny had been previously assessed [6]. Two of the predominant DGGE bands showed high similarity (> 99%) with the hydrogen-producing *Thermoanaerobacterium thermosaccharolyticum*. Members of the *Klebsiella, Bacillus *and *Sporolactobacillus *genera, detected in the inoculum sludge, were also predominant in R_gluc _and R_arab_.

### Effect of hydrogen partial pressure and pH on batch hydrogen production from arabinose and glucose

The effect of the hydrogen partial pressure, while using arabinose and glucose as substrates, was studied in batch experiments at pH 5.5 (equivalent to pH 5.0 at 70°C). Assays were performed allowing the accumulation of hydrogen in the headspace (no headspace flushing, NHF), or preventing hydrogen accumulation in the headspace (headspace flushing, HF). Subsequently, HF assays were performed at pH 7.0 (that is, pH 6.5 at 70°C) to study the effect of pH increase in hydrogen production. Substrates were added at the beginning of the experiment and a second addition was performed after complete depletion of the first load.

**Figure 5:**
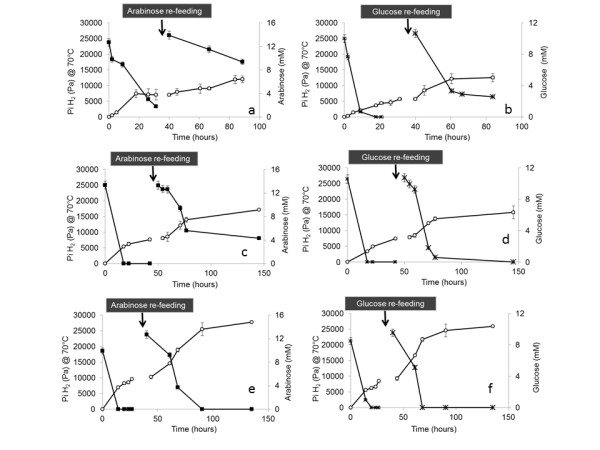
**Time course of hydrogen production and substrate consumption, O P_i_H_2 _; ■ arabinose; x glucose**. **a**, **b) **pH 5.5 without headspace flushing. **c**, **d) **pH 5.5 with headspace flushing. **e**, **f) **pH 7 with headspace flushing.

In the NHF (pH 5.5), maximum hydrogen concentration in the gas was achieved 44 and 20 h after the second addition of arabinose or glucose addition, respectively (Figure 5a, b). At this point, hydrogen partial pressure in both arabinose and glucose assays was roughly 1.2 × 10^4 ^Pa (at 70°C), which corresponds to a dissolved hydrogen concentration of 105 μM. From this point on, hydrogen production was not significant, even though only 35% of arabinose and 13% of glucose were present at the end of the experiment. Identical hydrogen yield, that is, 0.7 mol H_2 _per mole of substrate, was obtained for NHF arabinose and glucose experiments (Table 2).

**Table 2:** Substrate consumption and hydrogen yields from batchexperiments.

		Non Headspace Flushing (NHF)			
**pH**	**Substrate**	**Substrate consumed (%)**	**Yield (molH_2 _mol of substrate consumed^-1^)**	**Percentage of H_2 _produced from the theoretical yield** **(%)**	**pH at the end of the batch experiment**

5.5	arabinose	65	0.68 ± 0.05	20	5.8
	glucose	87	0.67 ± 0.13	17	5.2
	
		**Headspace Flushing (HF)**			

**pH**	**Substrate**	**Substrate consumed (%)**	**Yield (molH_2 _mol of substrate consumed^-1^)**	**Percentage of H_2 _produced from the theoretical yield** **(%)**	**pH at the end of the batch experiment**

5.5	arabinose	84	0.76 ± 0.06	23	5.3
	glucose	100	0.58 ± 0.07	15	5.2
7	arabinose	100	1.15 ± 0.03	35	6.5
	glucose	100	1.36 ± 0.14	34	6.8

Hydrogen production from arabinose could be increased in assays in which hydrogen partial pressure in the headspace was kept low (HF). A cumulative hydrogen production of 1. To -1.7 × 10^4 ^Pa (at 70°C) was attained in HF (pH 5.5) arabinose experiments (Figure 5c). This value is significantly higher than the one obtained in NHF experiments (*P*< 0.01: *t*-test), and corresponds to an increase of about 40% in hydrogen pressure. However, the highest increment in hydrogen cumulative production (that is, 62%) was observed in HF arabinose assays performed at pH 7.0 (cumulative hydrogen pressure of 2.8 × 10^4 ^Pa at 70°C (Figure 5f)). Arabinose was totally consumed in HF assays at pH 7.0, while a fraction substrate (approximately 1%) was not used in HF assays at pH 5.5 (Figure 5c, e). Nevertheless, non-consumed arabinose in HF at pH 5.5 was considerably lower than in NHF assays (Figure 5a, c). Hydrogen yields in HF arabinose experiments at pH 5.5 and pH 7.0 were 0.76 and 1.15 mol H_2 _per mole of substrate consumed, respectively (Table 2).

Hydrogen production values in HF and NHF glucose experiments at pH 5.0 were not significantly different. However, cumulative hydrogen production from glucose in HF experiments at pH 7.0 was significantly higher (*P *< 0.001: *t*-test) than at pH 5.5 (Figure 5d, f). Hydrogen cumulative pressure in HF glucose assays at pH 7 was of 2.6 × 10^4 ^Pa (at 70°C, Figure 5f). Glucose was totally consumed in HF assays both at pH 5.5 and pH 7.0. Hydrogen yields in HF glucose experiments at pH 5.5 and pH 7.0 were 0.6 and 1.4 mol H_2 _per mole of substrate consumed, respectively (Table 2).

**Figure 6:**
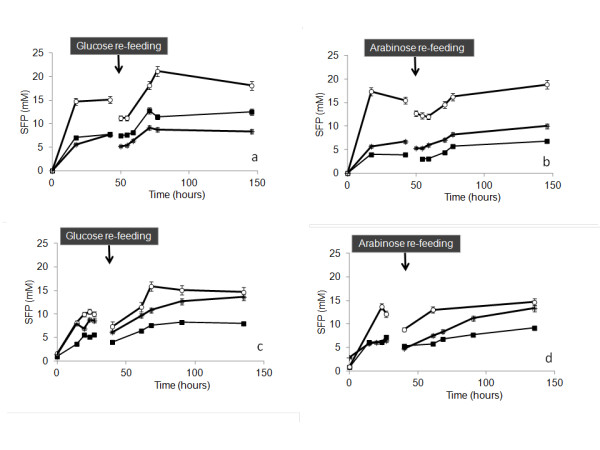
**Time course of soluble fermentation products, O ethanol; lactate; + acetate**. **a**, **b) **pH 5.5 with headspace flushing. **c**, **d) **pH 7 with headspace flushing.

At pH 5.5 approximately 20 mM of ethanol was produced from both substrates. At pH 7 ethanol formation did not exceed 15 mM (Figure 6). Acetate formation from both substrates at pH 7 achieved approximately 14 mM (Figure 6). In the case of glucose a decrease in 40% of lactate formation was also observed in incubations at pH 7.

## Discussion

### Continuous hydrogen production in EGSB reactors

R_arab _and R_gluc _showed similar performance during periods I and II of operation. However, when a higher organic loading rate was applied to the reactors (period III of operation, arabinose and glucose concentrations of 33.3 and 27.7, respectively), R_arab _showed a steady state hydrogen production rate 1.6 × higher than R_gluc_. Furthermore, hydrogen production rate measured in R_arab _was 1.3 × higher than the one reported by Abreu *et al. *[6] when feeding a EGSB reactor with a mixture of arabinose and glucose (1/1). Hydrogen production yield in R_arab _was 1.10 mol H_2 _per mole of arabinose consumed, which is considerably higher than the yields obtained in R_gluc _(0.75 mol H_2 _per mole of glucose) and R_gluc+arab _(0.77 mol H_2 _per mole glucose + arabinose) (Table 1). According to these results, the presence of glucose may possibly decrease the overall hydrogen yield in continuous operation, particularly when higher organic loading rates are applied. Lower hydrogen production observed in R_gluc _was likely associated with high lactate production (Figure 3). According to the Embden-Meyerhof pathway (Figure 1), sugar-derived pyruvate is (1) reduced to lactate, with regeneration of NADH (Table 3, reaction 1), or (2) oxidized to acetyl-CoA, with the production of reduced ferredoxin (Table 3 reaction (2)). Reaction (1) does not yield hydrogen, while in reaction (2) one mol of pyruvate results in the formation of 2 mol hydrogen. However, and considering Gibbs energy variations, reaction (1) seems to be energetically more favorable than reaction (2), especially at higher hydrogen partial pressures (Table 3).

**Table 3:** Gibbs free energy changes for some of the glucose and arabinose oxidation reactions.

Equation		ΔG^0^' *^a^* (kJ reaction^-1^)	ΔG' *^b^* (kJ reaction^-1^)
***Fermentative reactions***			
**NADH + H^+ ^+ pyruvate^- ^→ NAD^+ ^+ lactate^-^**	(1)	-25	
**2 ferredoxin(red) + 2H^+ ^→ 2 ferredoxin(ox) + H_2_**	(2)	+3	-25
***Glucose oxidation reactions***			
**1 glucose + 2H_2_O → 2 acetate^- ^+ 2CO_2 _+ 2H^+ ^+ 4H_2_**	(3)	-216	
**1 glucose → 1 butyrate^- ^+ 2CO_2 _+ 2H^+ ^+ 2H_2_**	(4)	-264	
**1 glucose → 2 lactate^- ^+ 2H^+^**	(5)	-197	
**1 glucose → 2ethanol^- ^+ 2CO_2 _+ 2H^+^**	(6)	-315	
***Arabinose oxidation reactions***			
**1 arabinose+ 1.67H_2_O → 1.67 acetate^- ^+ 1.67CO_2 _+ 1.67H^+ ^+ 3.33H_2_**	(7)	-192	
**1 arabinose → 0.83 butyrate^- ^+ 1.66CO_2 _+ 0.83H^+ ^+ 1.66H_2_**	(8)	-228	
**1 arabinose → 1.66 lactate^- ^+ 1.66H^+^**	(9)	-172	
**1 arabinose → 1.66 ethanol^- ^+ 1.66CO_2 _+ 1.66H^+^**	(10)	-269	

Standard Gibbs energies of formation of arabinose (in aqueous solution, pH 7 and 25°C) were estimated from the structures of the compounds, using a group contribution method described by [19]; standard Gibbs energies of formation of other compounds involved in the reactions were obtained from [20]

*^a ^*Gibbs free energies (at 25°C) calculated at standard conditions (solute concentrations of 1 M and gas partial pressure of 10^5 ^Pa).

*^b ^*Gibbs free energies (at 25°C) calculated at standard conditions (solute concentrations of 1 M and gas partial pressure of 1 Pa).

The fact that the microbial communities' composition in the reactors did not change along the three operational periods (Figure 4), suggests that the higher concentration of lactate produced in R_gluc _during period III is related to metabolic changes and is not a consequence of bacterial community shifts. Two of the predominant DGGE bands present in the reactors sludge could be affiliated with *Thermoanaaerobacterim thermosacharolyticum *(similarity higher than 99%). A draft genome of *T. thermosacharolyticum *(Joint Genome Institute) allowed a search of genes that encode metabolic enzymes involved in pyruvate conversion. A L-lactate dehydrogenase (EC 1.1.1.27) was present indicating the possibility of pyruvate reduction to lactate. Some genes codifying subunits of enzymes related to pyruvate-ferredoxin oxidoreductases and NADH oxidoreductases were also found but a complete picture of the mechanisms involved in pyruvate conversion to acetyl-CoA cannot be retrieved. Clones corresponding to other predominant DGGE bands present in reactors sludge exhibited highest sequence identity with *Klebsiella *sp. (99%) and *Bacillus coagullans *(99%). All these microorganisms are able to produce hydrogen and lactate, among other products, from a variety of carbon sources [21-23]. No genomic information is available for these species and physiological information is sometimes contradictory. For instance, the presence of *Bacillus coagullans *in hydrogen producing reactors has been associated to the increase of lactate production [24,25], but also to optimized hydrogen production [23,26].

The main possible reactions for the fermentation of arabinose and glucose, and the calculated Gibbs free energy of global reactions are shown in Table 3 (equations (3) to (10)) (only the reactions yielding experimentally detected soluble fermentation products in R_gluc _and R_arab _are represented). From a thermodynamic point of view, lactate formation from glucose and arabinose is less favorable than the formation of butyrate or ethanol. However, in continuous processes lactate was one of the main soluble fermentation products present in both reactors, especially in R_gluc _at higher influent, substrate concentration (27.7 mM). This might be related to the need of recycling reducing power from NADH. It has been proposed that thermophiles usually possess some escape routes to dispose of reductants in order to prevent obstructions in their metabolic flux. A possible route for this is the production of more reduced organic compounds like lactate, acetone and butanol [9,23]. A switch to lactate formation in *Thermoanaerobacterium *sp. was observed as a mechanism of reductant disposal and NAD(P)H oxidation [9,27].

### Hydrogen partial pressure and pH influence on hydrogen production yields

The metabolic pathways of hydrogen formation are sensitive to hydrogen concentrations and are subject to end-product inhibition. Results from this study showed that hydrogen production from arabinose and glucose is indeed higher when hydrogen is not allowed to accumulate in the headspace. Keeping low hydrogen partial pressure caused an increase in hydrogen production that could be mainly related to enhanced sugar utilization under these conditions. Nevertheless, in arabinose assays substrate was never completely depleted, not even when hydrogen was removed from the headspace. This can indicate that limiting factors other than *P*H_2_, such as liquid by-products inhibition, might be involved in hydrogen production from arabinose.

It has been reported that thermophilic hydrogen producing microorganisms could be inhibited by the presence of hydrogen, even when at very low partial pressure (from 0.1 × 10^4 ^to 7.5 × 10^4 ^Pa) [28]. Values of hydrogen partial pressure of 2 × 10^3 ^Pa, 1.6 × 10^3 ^Pa and 1.0 × 10^4 ^Pa were described as inhibitory for hydrogen production with *Thermotoga maritima, Pyrococcus furiosus *and *Caldicellulosiruptor saccharolyticus*, respectively [29]. In the present study hydrogen production by extreme thermophile mixed cultures using glucose and arabinose was inhibited at a *P*H_2 _similar to the one reported for C. *saccharolyticus *(that is, 1.2 × 10^4 ^Pa at 70°C).

Higher cumulative hydrogen production and yields were obtained at pH 7.0, either using glucose or arabinose as substrate. Lower hydrogen production at pH 5.5 was coupled to high ethanol and low acetate production (Figure 6). The present study suggests that, at extreme thermophilic conditions, maintenance of neutral pH (around 6.5 at 70°C) can aid preventing hydrogen losses by avoiding the production of more reduced organic compounds (such as lactic acid, acetone, butanol, and so on).

Overall, the results presented in this study show that both pH and hydrogen partial pressure affect hydrogen production efficiencies by extreme thermophilic mixed cultures. However, pH influenced hydrogen production in a greater extent than hydrogen partial pressure, both when using glucose or arabinose as substrate. Different soluble fermentation products' composition was observed in batch experiments and in continuous reactors. This can be related with the accumulation of soluble fermentation products happening in the batch assays, which can lead to different environmental conditions and, therefore, induce different metabolic pathways [30-32]. Nevertheless, batch results can give valuable insights for improving hydrogen production in continuous process.

## Conclusions

In continuous reactor, hydrogen production rate from arabinose was significantly higher than from glucose, when using organic loading rates of 14 KgCOD m^-3 ^d^-1^. This fact was associated with higher lactate production in the reactor fed with glucose, while in the arabinose-fed reactor, acetate and ethanol were the main end-products formed. The higher concentration of lactate was not a consequence of bacterial community shift, and is likely related to changes in the main metabolic pathways of glucose catabolism.

In batch mode, the effect of hydrogen partial pressure on hydrogen production from glucose was related to the extent of sugar utilization and not to the efficiency of substrate conversion to hydrogen. Furthermore, at pH 7.0, sugars uptake, hydrogen production and yield were higher than at pH 5.5, with both arabinose and glucose as substrates.

## Methods

### Continuous hydrogen production in EGSB reactors

Experiments were carried out in two plexi-glass EGSB reactors. An arabinose reactor (R_arab_) and a glucose reactor (R_gluc_) were fed with L-arabinose and glucose, respectively. EGSB reactors had a height of 1.95 m and internal diameter of 21 mm. Total liquid volume was 1.30 L, including reaction-zone volume of 0.7 L. Reactors were operated at 70 ± 1°C by means of an external water jacket, and pH inside the reactors was maintained at 5.5 ± 0.5. Superficial velocity was set at 10.0 m h^-1 ^(using internal recirculation) with an hydraulic retention time (HRT) of 9 h. Before start-up, R_arab _and R_gluc _were inoculated with 400 mL of granular sludge from a lab-scale hydrogen-producing reactor that had been fed with a mixture of arabinose (17 mM) and glucose (14 mM) for two months. Start-up of R_arab _was done using a constant arabinose feed concentration of 10.0 mM (period I); afterwards, arabinose concentrations of 16.6 mM (period II) and 33.3 mM (period III) were fed. Start-up of R_gluc _was done using a constant glucose feed concentration of 8.3 mM (period I); afterwards, concentrations of 13.8 mM (period II) and 27.7 mM (period III) were tested (Table 4). Arabinose and glucose concentration differed in order to have identical theoretical hydrogen yields in both reactors (that is, 33.3, 55.5 and 110.8 mM H_2 _for periods I, II and III, respectively). Sodium bicarbonate was added to the feed as alkalinity source (at a final concentration of 1 to 2 g L^-1^). Macronutrients solution containing 30 g L^-1^MgSO_4_.7H_2_O, 28.3 g L^-1 ^KH_2_PO_4 _and 170 g L^-1^) NH_4_Cl was also added (0.6 mL macronutrients solution per g of chemical oxygen demand (COD) in the feed).

**Table 4:** Operational conditions of glucose reactor (R_gluc_) and arabinose reactor (R_arab_)

Glucose Reactor (R_gluc_)		
**Feed Concentration (mM)**	**HRT (h)**	**OLR (Kg COD/m^3^/d)**
**8.3**	9	4.3
**13.8**	9	7.1
**27.7**	9	14.2

**Arabinose reactor (R_arab_)**		

**Feed concentration (mM)**	**HRT (h)**	**OLR (Kg COD/m^3^/d)**
**10**	9	4.3
**16.6**	9	7.1
**33.3**	9	14.2

### Batch experiments

#### Seed sludge

Granular sludge used for inoculating batch assays for studying arabinose- and glucose-conversion was collected from reactors R_arab _and R_gluc_, respectively.

### Medium composition and substrates

Assays were performed in 70 mL serum bottles containing 18 mL of buffered medium. Phosphate-buffered medium (20 mM) and bicarbonate-buffered medium (Stams *et al. *1993) were used for experiments at pH 5.5 and pH 7, respectively. Bottles with phosphate-buffered medium were flushed with N_2 _(100%), while bottles with bicarbonate-buffered medium were equilibrated with a mixture of N_2_:CO_2 _(80:20%). Both media were supplemented with trace elements, salts and vitamins according to the procedure described by Stams *et al. *[33]; yeast extract was added to a final concentration of 0.5 g L^-1^. Medium was reduced with 0.8 mM sodium sulfide (Na_2_S.9H_2_O) and inoculated with 0.4 g of granular sludge. Arabinose (13 mM) and glucose (11 mM) were used as the main carbon source. Bottles were incubated in the dark at 70°C without shaking. After substrate depletion, a second pulse of 13 mM arabinose or 11 mM glucose was added and incubation extended.

### Effect of hydrogen partial pressure

The effect of hydrogen partial pressure on hydrogen production from arabinose and glucose was investigated in batch mode at pH 5.5. Two series of batch experiments were performed: in series NHF (no headspace flushing), hydrogen was allowed to accumulate in the gas phase, while in series HF (headspace flushing) hydrogen was removed from the bottles' headspace and replaced by 100% N_2_. All experiments were performed in triplicate and included controls without substrate. Sugars consumption, production of hydrogen gas and soluble fermentation products were monitored. Dissolved hydrogen concentration was calculated using the Henry's law at 70°C: K_H_*Pi, where K_H _is the Henry's law constant for hydrogen (8.7 × 10^-9 ^M/Pa at 70°C).

### Effect of pH

The effect of pH on hydrogen production from arabinose and glucose fermentation was studied in two series of batch experiments, one at pH 7.0 and the other at pH 5.5. Incubation was done at 70°C and all the experiments were performed in triplicate. Sugars consumption, formation of hydrogen gas and soluble fermentation products were monitored and dissolved hydrogen concentration was calculated using the Henry's law at 70°C.

### Analytical methods

Hydrogen concentration in the gas phase was determined by gas chromatography (GC) using a Hayesep Q column (80/100 mesh) and thermal conductivity detector Varian 3300 Gas Chromoatograph, (Varian, Walnut Creek, USA)) with nitrogen (30 mL minute^-1^) as the carrier gas. The injector, detector and column temperatures were 120, 170, and 35°C respectively. Methane and carbon dioxide content of the gas phase from batch experiments and EGSB reactors was determined by gas chromatography using a *Porapack Q *(100 to 180 mesh) column, with helium as the carrier gas at 30 mL minute^-1^, and a thermal conductivity detector. Temperatures of the detector, injector and oven were 110, 110 and 35°C, respectively. In the EGSB reactors gas flow rate was measured by a *Ritter Milligascounter *(Dr. Ing. Ritter Apparatebau GmbH, Bochum, Germany). Volatile fatty acids (VFA), ethanol, lactic acid, L-arabinose and glucose were determined by high performance liquid chromatography using an HPLC (Jasco, Tokyo, Japan) with a *Chrompack column *(6.5 × 30 mm^2^); sulfuric acid (0.01 N) at a flow rate of 0.7 mL minute^-1 ^was used as mobile phase. Column temperature was set at 60°C. Detection of VFA, lactic acid, ethanol, arabinose, glucose was made sequentially using a UV detector at 210 nm and a RI detector.

### PCR-DGGE

Representative granular sludge samples were collected from R_arab _and R_gluc _and stored at -18°C. Total genomic DNA was extracted from approximately 500 μL of sample by using the FastDNA SPIN kit for soil (Qbiogene, Carlsbad, CA, USA). 16S rRNA gene fragments of approximately 450 bp were amplified for DGGE analysis by PCR using a Taq DNA polymerase kit (Life Technologies, Gaithersburg, MD, USA) using the primer set 954GC-f and 1369-r, as previously described by Nubel *et al. *[34]. The size of the obtained PCR products was checked by comparison with appropriate size and mass standard (MBI Fermentas, Vilnius, Lithuania), by electrophoresis on an 1% (w/v) agarose gel and ethidium bromide staining. Gels ran at a constant voltage of 100 V in an agarose gel electrophoresis system (Mupid-EX, Seraing, Belgium). Nucleic acids were detected using an UV transilluminator (BioRad, Hercules, CA, USA).

DGGE analysis of the amplicons was done by using the DCode system (Bio-Rad). PCR products were electrophoresed in a 0.5 × Trisacetate-EDTA buffer for 16 h at 85 V and 60°C on polyacrylamide gel (8%) containing a linear gradient ranging from 30% to 60% denaturant. Silver staining of DGGE gels was performed as previously described [35]. DGGE gels were scanned at 400 dpi and the DGGE profiles compared using the Bionumerics 5.0 software package (Applied Maths, Gent, Belgium). Similarity indices (Si) of the compared profiles were calculated from the densitometric curves of the scanned DGGE profiles by using the Pearson product-moment correlation [36].

### Gibb's Free energy calculations

Standard Gibb's free energy at 25°C (ΔG^o^) was calculated using standard Gibb's free energy of formation values (ΔG^o^_f_) obtained from the literature [19,20] or calculated using the group addition method [19].

## Abbreviations

DGGE: denaturing gradient gel electrophoresis; EGSB: expanded granular sludge bed; NADH: reduced form of nicotinamide adenine dinucleotide; PCR: polymerase chain reaction; *P*H_2 _: hydrogen partial pressure.

## Competing interests

The authors declare that they have no competing interests.

## Authors' contributions

All authors contributed intellectually via scientific discussions during the work and have read and approved the final manuscript. AAA designed the study, executed the experimental work, data interpretation and drafted the manuscript. IA and DK commented on the manuscript and contributed to the design of the study. DZS participated in data interpretation, reaction thermodynamics calculations and reviewed the manuscript. MMA contributed to the design of the study, data interpretation and reviewed the manuscript. All authors read and approved the final manuscript.
